# Combination of lenalidomide and vitamin D enhances MOR202-mediated cytotoxicity of macrophages: It takes three to tango

**DOI:** 10.18632/oncotarget.26531

**Published:** 2019-01-01

**Authors:** Cindy Flamann, Leonhard Busch, Andreas Mackensen, Heiko Bruns

**Affiliations:** Heiko Bruns: Department of Internal Medicine 5-Hematology/Oncology, Friedrich-Alexander University Erlangen-Nürnberg, Erlangen, Germany

**Keywords:** myeloma-associated macrophages, myeloma, CD38, antibody-dependent cellular phagocytosis, vitamin D

Although multiple myeloma (MM) has become more treatable in the past decade by application of proteasome inhibitors (PI) and immunomodulatory drugs (IMiDs) [1], there is an urgent need for development of novel treatment alternatives. Monoclonal antibodies (mAbs) have become of growing interest as promising approaches over the last few years. Since rituximab, an anti-CD20 mAbs, is efficiently established in the treatment of B-cell NHL, previous attempts for application of this mAbs in MM patients have been rather unsuccessful [2]. However, several potential surface targets for therapeutic antibodies have been evaluated. Especially the type II single-chain transmembrane glycoprotein CD38, which is highly expressed on multiple myeloma cells, seems to be a promising candidate for immunotherapy of MM. The human monoclonal antibody MOR202 directed against CD38 undergoes currently clinical trials for treatment of relapsed or refractory MM [3]. Although anti-CD38 antibodies have shown substantial activity as monotherapy, observations from preclinical studies and recent clinical trials indicate that combination with IMiDs such as lenalidomide further enhances their anti-MM efficacy [4].

Macrophages mediate antibody-dependent cellular phagocytosis (ADCP) and antibody-dependent cell-mediated cytotoxicity (ADCC) but also serve in a MM-supportive role as so-called myeloma-associated macrophages (MAMs) [5]. Despite their clear abundance in close proximity to MM-cells, little is known of their potential role in mediating the therapeutic antibody effects and whether or how lenalidomide modulates their function.

Interestingly, vitamin D plays a key role in regulating effector functions of human macrophages [6], and vitamin D deficiency is also extremely common in MM. The beneficial effects of vitamin D on macrophages are closely linked to the expression of the vitamin D-1-hydroxylase CYP27B1, which catalyzes the conversion of 25-OH-hydroxy-vitamin D3 (25D3) to the bioactive 1,25-di-hydroxy-vitamin D3 (1,25D3). Active 1,25D3 binds to the vitamin D receptor (VDR) and induces a variety of target genes containing vitamin D response elements (VDRE) in their promoters. In human macrophages, 1,25D3 induces central tumoricidal killing mechanisms. In particularly those, which are important co-mediators for the therapeutic effects of antibodies, such as phagocytosis, expression of antimicrobial peptides and superoxide formation [6] [7].

During the last years, we have been focusing on the tumoricidal effector functions of macrophages. Although macrophages can, in principle, target neoplastic cells and mediate ADCP, tumor-associated macrophages (TAMs) regularly fail to exert direct cytotoxic functions. The underlying mechanisms responsible for this observation remain unclear. Since vitamin D acts as a key regulator of the human macrophages’ effector functions, as well as the finding that vitamin D deficiency negatively affects treatment of high-grade NHL patients with rituximab containing chemotherapy [8], we speculated that effector functions of TAMs are substantially determined by vitamin D.

We observed that MAMs (as compared to bone marrow macrophages of healthy donors) exhibit a defective vitamin D metabolism with reduced expression of CYP27B1 [9]. As a consequence, MAMs cannot convert 25D3 into bioactive 1,25D3, and were unable to release 1,25D3 into the local tumor microenvironment. In order to tackle the question whether vitamin D affects the MM cells, we performed chromatin immunoprecipitation (ChIP) analysis of endogenous VDR in MM cells in the presence and absence of 1,25D3. In this context, we were able to show that the promotor region of CD38 in MM cells contains VDR binding sites, and that treatment with 1,25D3 induces an upregulation of CD38 on primary MM cells. In line with these observations, the increased CD38 expression on MM cells resulted in enhanced binding of MOR202 to the surface of MM cells after incubation with 1,25D3. Given that the efficacy of immunotherapy with CD38-targeting antibodies depends not only on the tumoricidal activity of effector cells, but also on the expression levels of their target, vitamin D fulfills two major objectives: Increasing effector function of macrophages and increasing the vulnerability towards anti-CD38 antibodies by enhancing CD38 expression on MM cells. Considering the key role of the vitamin D pathway in regulating effector functions of macrophages and expression of CD38 on MM cells, an attractive therapeutic approach would be to restore the vitamin D pathway in MM patients in order to promote ADCC/ADCP. We found, that IMiDs, in particular lenalidomide and pomalidomide, were able to enhance CYP27B1 expression in macrophages of MM patients under IMiDs treatment. The augmented CYP27B1 expression leads to an increased conversion of 25D3 into bioactive 1,25D3 and to an improvement of the MOR202-mediated cytotoxicity of MAMs. Moreover, the induced CYP27B1 activity and the enhanced 1,25D3 production were required for the observed lenalidomide-triggered MOR202 mediated cytotoxicity of macrophages.

We and others have previously shown, that vitamin D promotes the macrophages’ tumoricidal potency against lymphomas and improves the efficacy of rituximab-dependent cytotoxicity [6]. In fact, recent clinical studies have shown that vitamin D deficiency was associated with reduced event-free survival and overall survival in DLBCL and follicular lymphoma (FL) patients receiving rituximab [8, 10]. Strikingly, these findings have resulted in recommendation to supplement vitamin D before and during chemo-immuno-therapy. Since MAMs display a defective vitamin D pathway, and they can therefore not react adequately on this vitamin D supplementation, it should be considered to restore the vitamin D pathway simultaneously in these patients by IMiDs.

**Figure 1:**
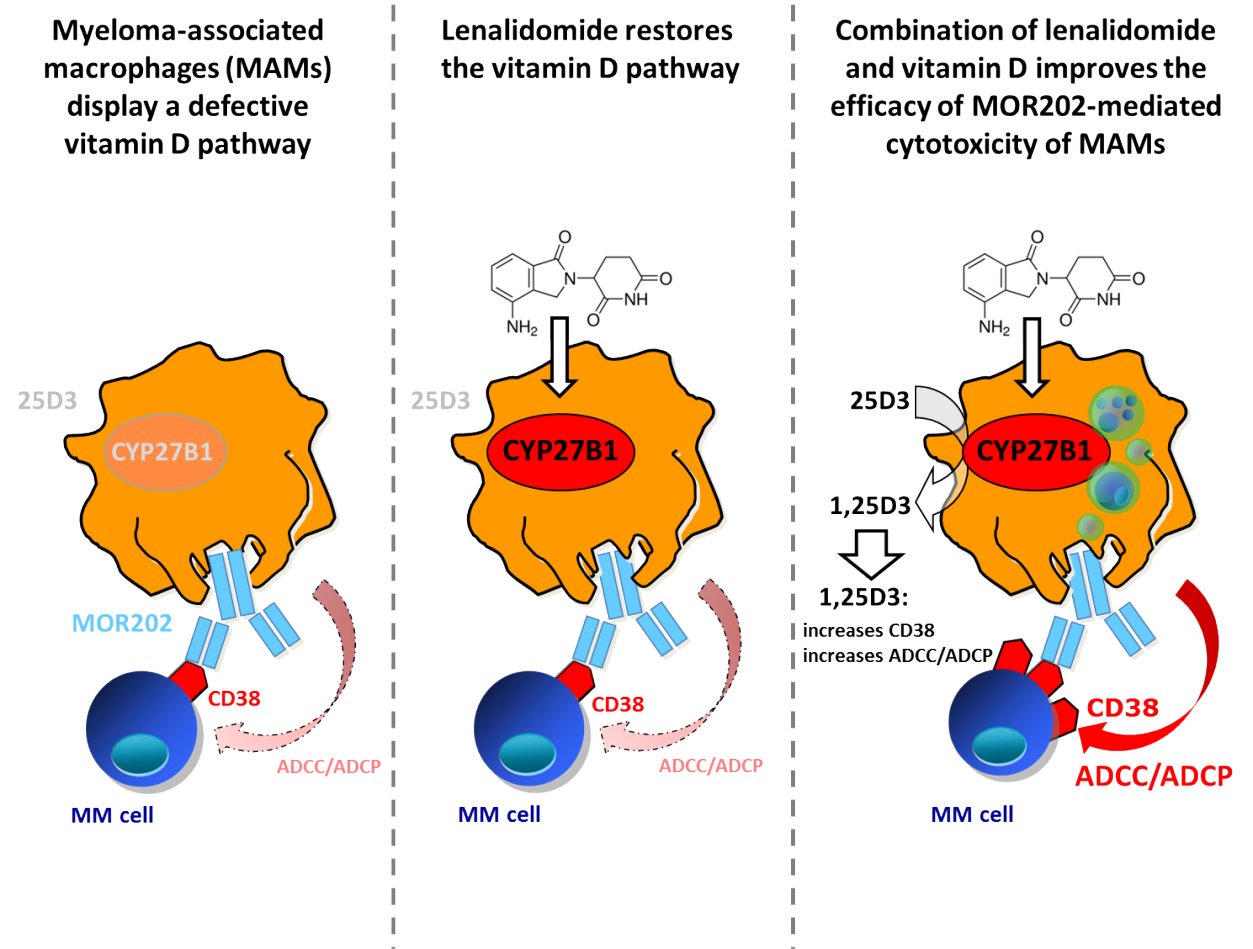
(from left to right): Myeloma-associated macrophages (MAMs) regularly fail to exert MOR202-mediated cytotoxicity against MM cells, due to their impaired vitamin D metabolic pathway and reduced expression of CYP27B1 This reduced CYP27B1 expression can be overcome by lenalidomide, which significantly increases the expression of CYP27B1, leading to an increased conversion of 25D3 into bioactive 1,25D3. Increased 1,25D3 level promote CD38 target expression on MM cells and combination of lenalidomide and exogenously added 25D3 improves the efficacy of MOR202-mediated cytotoxicity of MAMs.

Taken together, it may be postulated that reconstitution of the vitamin D metabolism in MAMs by lenalidomide and constant vitamin D supplementation of MM patients improves the therapeutic efficacy of anti-CD38 mAbs like MOR202 (Figure 1). Our findings could thereby provide one mechanistic explanation for the clinical observation that combination of anti-CD38 mAbs with IMiDs such as lenalidomide further enhances their anti-MM efficacy [4].
